# Palliative care in intensive care units: why, where, what, who, when, how

**DOI:** 10.1186/s12871-018-0574-9

**Published:** 2018-08-16

**Authors:** Sebastiano Mercadante, Cesare Gregoretti, Andrea Cortegiani

**Affiliations:** 1 Anesthesia and Intensive Care Unit and Pain Relief and Supportive-Palliative Care Unit, La Maddalena Cancer Center, Via san Lorenzo 312, 90145 Palermo, Italy; 2 Department of Biopathology and Medical Biotechnologies (DIBIMED). Section of Anestesia, Analgesia, Intensive Care and Emergency. Policlinico Paolo Giaccone, University of Palermo, Via del vespro 129, 90127 Palermo, Italy

**Keywords:** Palliative care, Intensive care unit, ICU, Patient-centered care, End-of-life care

## Abstract

Palliative care is patient and family-centered care that optimizes quality of life by anticipating, preventing, and treating suffering when “curative” therapies are futile. In the Intensive Care Unit (ICU), critically ill patients receive life-sustaining therapies with the goal of restoring or maintaining organ function. Palliative Care in the ICU is a widely discussed topic and it is increasingly applied in clinics. It encompasses symptoms control and end-of-life management, communication with relatives and setting goals of care ensuring dignity in death and decision-making power. However, effective application of Palliative Care in ICU presupposes specific knowledge and training which anesthesiologists and critical care physicians may lack. Moreover, logistic issues such protocols for patients’ selection, application models and triggers for consultation of external experts are still matter of debate.

The aim of this review is to provide the anesthesiologists and intensivists an overview of the aims, current evidence and practical advices about the application of palliative care in ICU.

**Electronic supplementary material:**

The online version of this article (10.1186/s12871-018-0574-9) contains supplementary material, which is available to authorized users.

## Background

The aim of intensive care is the maintenance of vital functions to reduce mortality and prevent morbidity in patients with a severe critical illness. Despite the development of new technologies and the improvement of care, death rate in the intensive care unit (ICU) remains high [1, 2], ranging 20–35%, with variations according geographical regions. Mortality rate was higher in upper-middle income countries than to ICUs in low and lower-middle or high-income [3]. In the latest years, ICU admissions in the last month of life have been growing up to 30% [1, 4]. When the organ dysfunction of critical illness does not respond to treatment, and the goals of care cannot be achieved anymore, or when life support becomes to be non proportional to expected prognosis, ICU physicians should provide an acceptable death [5, 6]. When life-sustaining therapies are unable to meet the patient’s goals, or paradoxically may result to be more burdensome than beneficial, withdrawal and withholding of therapies is a commonplace among ICU physicians [7]. In general, dying patients lack decision-making capacity. Advanced directives, when available, should guide the decision-making process, although it is often a medical team decision. This process may be complex and emotionally draining. Physician training in graduate and continuing medical education may provide guidance and support. Moreover, complications of polypharmacy, no realistic overview, poor attention for quality of life, and communication with relatives, are of concern. However, it is often difficult for physicians to initiate an appropriate discussion with patients’ relatives. Thus, ICU clinicians require knowledge and competence on the many aspects of withholding/withdrawing interventions and, in general, on end of life supports [7–11], including adoption of some treatment limiting the suffering, good communication with relatives, and how to afford some ethical issues. The use of sedatives, analgesics, and other non-pharmacological methods to ease distressing symptoms, as well as careful communication to support the decision-making process, including autonomy, capacity determination, and surrogacy, are of paramount importance, even during the phase of active treatments [12, 13]. Moreover, ICU staying is also an unpleasant experience. Many symptoms commonly encountered in palliative care practice, such as pain, thirst, anxiety, sleep disturbances and dyspnea, frequently develop in critically ill patients [14–18]. These symptoms may persist even after ICU discharge and may produce a post-intensive care unit syndrome, with cognitive, psychiatric and physical consequences [19–21]. Potentially, ICU clinicians should anticipate this approach to mitigate these problems [22]. Patients’ families may experience psychological and physical distress, including depression, fear, anxiety, fatigue, anorexia, and early posttraumatic stress symptoms [23], and the persistence of symptoms after ICU can result in a “post-intensive care unit syndrome-family” in bereaved families [24–26]. Finally, ICU clinicians are at risk for emotional and psychological distress when facing these situations. Additional file 1 depicts critical care physician’s doubts when a curative plan seems no longer effective.

Palliative care physicians are traditionally involved in issues regarding end of life care. Palliative care is patient and family-centered care with the aim of optimizing quality of life by anticipating, preventing, and treating suffering, providing a comprehensive management of patients facing incurable diseases, regardless of age, diagnosis, or prognosis. Moreover, palliative care focuses issues including symptom distress (physical, psychological, spiritual), capacity to communicate and share decision-making, while reducing the burden for caregivers [9, 27] . Apparently, palliative care and intensive care may be the opposite ends of care; the former is considered as “talking medicine” and the latter as “technical medicine.” However, there are commonalities between modalities of care, as both fields may overlap in virtuous circle to provide the best benefit for ICU patients. As a merger of approaches and cultures begin to seem natural, and collaborative opportunities are consequential, this review will examines some points regarding the apparent oxymoron existing between ICU and palliative care.

### Why

Unrelieved and distressing symptoms are present for most ICU patients. In a prospective study of ICU patients at high risk of dying, distressful symptom prevalence ranged from 27 to 75%. Delirium was found in about one-third of patients who could be evaluated [15]. Similarly, relatives showed a high level of symptom distress, with 57% having traumatic stress, 70–80% anxiety and depression, other than physical and emotional symptoms, particularly in the youngest [28]. These findings suggest that a palliative care assessment should be started as early as possible to allow more focused interventions to anticipate or minimize unnecessary suffering. In a retrospective study using medical record review and surveys of family members, it has been found that certain whole-person and preparation-for-death aspects of the dying process, and avoidance of aggressiveness at the end-of-life care, were more likely to be associated to better quality ratings. This finding suggests that care at the end of life in the ICU should not include managing pain and symptoms only, but also supporting death dignity and respect, which are typical issues necessitating palliative care experience [29]. It has been estimated that 14–20% of ICU patients meet the typical “triggers” for palliative care consultation [30]. Proactive palliative care involvement on ICU rounds for high-risk patients has been found to be associated with earlier ICU family meetings and shorter hospital length of stay [31]. Of interest, although few specific interventions were found to increase family satisfaction in ICU, good-quality communication, support for shared decision-making, and specific patient-care measures were associated with increased satisfaction with end-of-life care [32]. Thus, principles of palliative care need to be mandatorily applied in ICUs.

### Where

Potentially, patients who are unfit for further aggressive treatments, could be transferred to a specialized palliative care setting, where there are physicians having certification in hospice and palliative medicine, and withdrawal procedures are commonly used in end-of-life care patients.. This modality has not been reported properly and is difficult to organize, also because hospice care is often offered in extra-hospital settings. Acute palliative care units may facilitate the process of transition of care. For instance, successful withdrawal of ventilatory support and a natural death at home were possible with a logistic support from the hospice organization and the expertise of the hospice team [33].

In a modern view, the principal domains of palliative care, including relief of distressing symptoms, effective communication about care goals, patient-focused decision-making, caregiver support, and continuity across care settings, should be performed in ICU [6, 9, 34].

### What

Many issues are included in the content areas of palliative care. The management of physical and psychological symptoms, as well as spiritual and existential distress, prognostication, communication about care goals in relation to patient values and preferences are fundamental. Thus, a proactive identification of problems, an early sharing decision making with relatives, prospecting an advance care planning and possible scenarios for end of life decisions, are of paramount importance in ICU.

Moreover, ethical and legal aspects of decision making, transition planning, care during the dying process, and family support including grief and bereavement care complete the pattern of palliative care competencies. The goal-setting with a family experiencing high levels of distress or conflicts among family members, or supporting a bereaved family represent a typical examples of clinical changes [35].

Another relevant issue regards palliative sedation in the dying patients with high levels of terminal suffering. Some refractory symptoms may require to lower the level of consciousness by proportionate doses of hypnotics. Of interest, either the modality and the intent, are different from most sedation performed in ICU.

### Who

Early efforts of palliative care in ICU focus on improving the quality of dying and death. However, its scope extends more broadly to meet the needs of patients affording life-supporting therapies and expected to survive. The general palliative care issues should be responsibility of all ICU clinicians, who need a basic knowledge and skills for symptoms management, appropriate techniques of communication, capability in sharing decision-making based on patients’ values, goals and preferences (named also “integrative model”) [34, 36]. Indeed, palliative care specialists can provide essential input in ICU. Although symptom management was the most common reason for palliative care consultation (named also “consultative model”), symptom assessment was infrequently documented. Furthermore, palliative care consultants performed better in offering spiritual support and managing documented symptoms [37]. Regrettably, the workforce of such specialists will remain inadequate to meet increasing needs in ICU population. For such reasons, it has been recommended a mixed model, where primary palliative care of ICU physicians is combined with specialist palliative care physicians.

### When

Life-prolongation and palliation can be seen as dichotomous aspects of care. However, ICU clinicians and palliative care physicians both care critical and life-threatening conditions. Although the aims of palliative care and critical care may initially seem divergent, values and goals in critical care and palliative care are similar, as saving or prolonging life may conciliate with alleviating suffering and improving quality of life, and death. Of course, the primary goal of each discipline is the secondary goal of the other. One should consider that palliative care originated as end-of-life care in the 1960s [38]. However, since then its meaning and scope have expanded far beyond its roots. According to the World Health Organization [39], and a more recent and broader definition, the goal of palliative care is “to maintain and improve the quality of life of all patients and their families during any stage of life-threatening illness”. Palliative care aims to prevent and relieve suffering by early identification, assessment, and treatment of physical and psychological symptoms, as well as emotional, and spiritual distress [39]. All patients receiving curative treatments should receive palliative care simultaneously and individually according to the patient’s and family’s needs and preferences [6]. Discussions about the changes of goals of care and a more proportional level of treatment should be started early in the ICU [40, 41]. During the course of ICU admission, frequent meetings with relatives are mandatory as the ICU patient’s condition evolves. Life-support treatments should be systematically re-evaluated to determine if the care plan is achieving its goals. Physicians should facilitate these discussions, which should take place in a private and personal environment, involving patients’ surrogates, and team members. Sharing opinion facilitates the decision-making process. Thus, palliative care assessment should be performed early during ICU admission, anytime typical issues for a palliative care evaluation emerge.

### How

Basic symptom management and discussion of goals of care in relation to the patient’s prognosis and preferences, are the core aspects of palliative care, and should be part of routine ICU practice, thus within the competency of any ICU clinician [8]. Decision-making on withholding and withdrawal of life-sustaining therapies in ICU is not homogeneous worldwide. This process depends on several factors such as legal, political, religious issues other than experience and patients’ characteristics [42]. The capacity of withdrawal or withholding aggressive and futile treatments should belong to the armamentarium of any ICU clinician [43, 44]. For example, in withdrawal of mechanical ventilation, terminal extubation resulted to be a practice largely approved by family members, allowing relatives to be present at time of death, in comparison with terminal weaning. Of interest, no differences in length of stay and doses of sedatives were found [45].

End-of-life protocols seem to be effective in achieving adequate patient comfort in ICU. Few signs of distress were reported in most patients in whom life-sustaining measures were withdrawn. The use of opioids and sedatives increased significantly during treatment withdrawal but did not contribute to hasten death. Opioids are often successfully given to patients undergoing terminal withdrawal of mechanical ventilation [10, 13, 46]. Of interest, opioid doses were influenced by the level of previous opioid consumption (namely opioid-tolerance), and higher doses were associated with a longer time to death [47]. Thus, dying without distressing signs is practically possible and ethically feasible, as this does not hasten death [48, 49].

There are two main models for ICU–palliative care integration. The “integrative model” seeks to embed palliative care principles and interventions into daily practice by the ICU team for all patients and families facing critical illness. In some circumstances an ICU physician might wish to obtain expert contributions from a palliative care team, as more advanced palliative care skills and interprofessional expert input may be necessary to face determined situations. The “consultative model” focuses on increasing the involvement of palliative care consultants in the care of ICUs patients identified as at highest risk for poor outcomes. This model provides and improves palliative care quality in the ICU. Of interest, other palliative care providers, including nurse practitioners, spiritual caregivers, and social workers could be involved. Psychological, social, and spiritual domains are relevant for the care of patients and their relatives.

The integration of palliative care experts in ICUs is of benefit to patients, families, and critical care clinicians. After palliative care consultation, 29% of patients were disconnected from mechanical ventilators, about 16% of patients discontinued the use of inotropic support, 15.3% stopped artificial nutrition, 6.4% stopped dialysis, and 2.5% discontinued hydration. Recommendations on pain and symptom management were made for 51% and 52% of patients. In this preliminary study there was an increase in the rate of the formalization of advance directives, with 33% of the patients having ‘do not resuscitate’ orders in place prior to consultation, and 83.4% after the intervention. Of interest, the involved team referred half of patients to hospice. Median survival times were not significantly different [50]. Preliminary evidence suggest that such models may be associated with improved quality of life, higher rates of formalization of advance directives and utilization of hospices, as well as lower use of certain non-beneficial life-prolonging therapies for patients who are at the end of life. Indeed, in the integrative model, palliative care principles are incorporated as part of routine practice by ICU physicians.

Each model can be successful in delivering palliative care in ICU, according to the resources and needs of individual ICUs [34]. Some points are in favour of the integrative model when workforce shortages may limit dependence on palliative care specialists, at least in the short term [30]. Moreover, reliance on specialty palliative care could undermine the therapeutic relationship with patients and relatives, giving the impression of fragmented care [51]. Finally, an external consultation may reduce the needs for ICU clinicians to develop palliative care knowledge and skills. On the other hand, specific situations often benefit from a palliative care specialist, when palliative care has not already been integrated among ICU physicians [9].

The mixed model includes both options [52]. The treating specialist could manage more relevant palliative care problems in combination with a palliative care specialist [35], as it occurs with other specialists to address peculiar problems arising in the clinical setting of critically ill patients. Criteria used to screen patients for unmet palliative care needs can trigger a palliative care consultation, while prompting care processes implemented by the ICU team itself [36]. Of interest, it has been reported that a palliative care consultation was associated with more frequent “do not resuscitate” code status and hospice referrals, hospital length of stay and direct cost reductions. Thus “trigger” programs in the ICU may improve utilization of palliative care services, improve patient comfort, and reduce invasive, often futile end-of-life care [53].

To meet palliative needs of critically ill patients and families, it will be relevant both to increase the capability of ICU clinicians to provide basic palliative care and to expand the specialist palliative care workforce. Clinical experience and a growing body of evidence suggest that palliative goals can improve the quality of care in critical ill patients [51]. Evidence regarding the use of palliative care in ICU, however, remains poor. Outcome measures were heterogeneous among study designs and many studies utilized different outcome measures, sometimes stratified between decedents and survivors. Because of the wide variation in outcome measures, study comparison was challenging. The heterogeneous study findings prevalently reported a decrease in ICU and hospital length of stay [51].

## Conclusion

Although ICU and palliative care may seem to be polar opposites, they share fundamental features. In terms of evidence, ICU-based palliative care interventions are difficult to evaluate, due to the heterogeneity of studies. Existing data, however, suggest that proactive palliative care in the ICU, using both model of intervention, either consultative or integrative, may decrease hospital and ICU staying and do not affect mortality [31, 51]. Barriers to implement this approach include misperception of critical care and palliative care as sequential processes rather than complementary and simultaneous approaches, unrealistic expectations, and concerns that palliative care may hasten death. This misleading concepts are the consequence of insufficient training in communication and palliative care skills [52]. Indeed, a project framework and recommendations can be effectively used to increase the number of palliative care consults in the ICU and anticipate this kind of evaluation [54]. Daily pre-rounds between the palliative care and ICU teams increased palliative care services for patients at risk for poor outcomes, who may benefit from a palliative care consultation. Palliative care consultation increased significantly from 5 to 59% for patients who died in ICU during the intervention period [55].

ICU physicians should be competent in all aspects of terminal care, including the practical and ethical aspects of withdrawing different modalities of life-sustaining treatments and the use of pharmacological and non-pharmacologic approaches to limit the suffering of the dying process. A recent national survey with a large number of respondents suggested that training in palliative care and its clinical application need to be strongly implemented [56]. Many scientific organizations are encouraging the development of palliative care clinical programs locally in their hospitals and health care organizations [7, 57]. ICU physicians should become familiar with such issues and ICU doctors should get a specific dedicated training in palliative care principles. Enhanced professional education and training in palliative care are the means for the necessary changes to ensure that all critically ill patients and their families have access to an excellent level of palliative care able to successfully meet their needs.

## Additional file

Additional file 1: Critical care physician’s most frequent questions about palliative care in ICU. The figure depicts critical care physician’s doubts facing a clinical picture of a patient where the curative plan seems no longer effective. The questions describe most frequent open question about palliative care in ICU. (TIFF 4303 kb)

## Abbreviations

ICU: Intensive Care Unit
PICS: Post-intensive care unit syndrome
