# Supplementary figures and images for: Palliative care in intensive care units: why, where, what, who, when, how
Source: BMC Anesthesiol. 2018 Aug 16;18:106. doi: 10.1186/s12871-018-0574-9 (PMC6094470; doi:10.1186/s12871-018-0574-9)

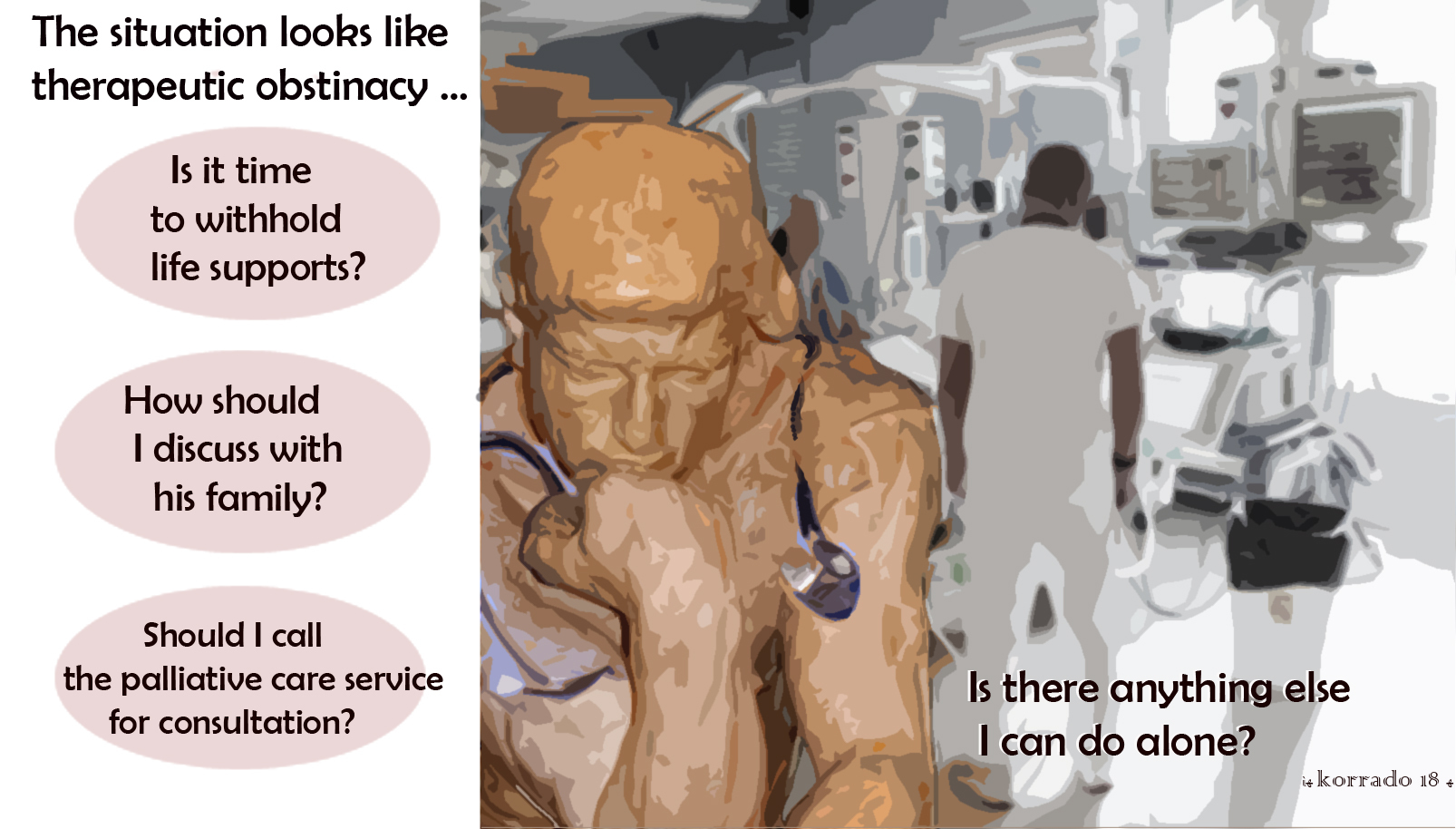

Supplement: Supplementary file 1 — Critical care physician’s most frequent questions about palliative care in ICU. The figure depicts critical care physician’s doubts facing a clinical picture of a patient where the curative plan seems no longer effective. The questions describe most frequent open question about palliative care in ICU. (TIFF 4303 kb) [file 12871_2018_574_MOESM1_ESM.tiff]
